# Data on optical microscopy and vibrational modes in Diphenyl Dinaphthothienothiophene thin films

**DOI:** 10.1016/j.dib.2019.104522

**Published:** 2019-09-16

**Authors:** Yoshiaki Hattori, Yoshinari Kimura, Takumi Yoshioka, Masatoshi Kitamura

**Affiliations:** Department of Electrical and Electronic Engineering, Kobe University, 1-1, Rokkodai-cho, Nada-ku, Kobe, 657-8501, Japan

**Keywords:** DPh-DNTT, Raman spectroscopy, Vacuum deposition, 2D island, Fractal

## Abstract

The thin-films of 2,9-diphenyl-dinaphtho[2,3-*b*:2′,3′-*f*]thieno[3,2-b]thiophene (DPh-DNTT) prepared by vacuum deposition was observed by the optical microsope. By applying the dark-field mode in observation and/or image processing after imaging appropriately, morphological structure with a resolution of a few nanometers height was visualized easily and quickly. The technique can be used in a similar to atomic force microscopy, which is commonly used for imaging surface morphology. Moreover, the vibrational modes of a DPh-DNTT molecule calculated by quantum chemistry program is described as well as the comparison of the experimental Raman spectra for identification. The presented data are produced as part of the main work entitled “The Growth Mechanism and Characterization of Few-layer Diphenyl Dinaphthothienothiophene Films Prepared by Vacuum Deposition” (Hattori et al., 2019).

**Table:** Specifications Table

Subject	Surfaces, Coatings and Films
Specific subject area	Organic films
Type of data	Table, Image, Figure
How data were acquired	Optical microscope (LV100, Nikon) with a digital camera (EOS Kiss X4, Canon) Objective lenses (LU Plan Apo 150x/0.90, Nikon) Micro-Raman spectrometer (NRS-7100, Nihon Bunko) Quantum chemical calculations (Gaussian 09 program package)
Data format	Raw, Analyzed
Parameters for data collection	The thin films were deposited on Si substrates with thermally grown 90-nm-thick SiO_2_ at a pressure of the order of 10^−4^ Pa with a deposition rate of 0.05 Å/s. The micro-Raman spectroscopy was performed with a 532-nm laser (5.3 mW).
Description of data collection	The thin-films were characterized by the optical microscope and the micro-Raman spectrometer. Quantum chemical calculations were performed using the Gaussian 09 program package.
Data source location	Kobe University
Data accessibility	With the article
Related research article	Yoshiaki Hattori, Yoshinari Kimura, Takumi Yoshioka, and Masatoshi Kitamura, The Growth Mechanism and Characterization of Few-layer Diphenyl Dinaphthothienothiophene Films Prepared by Vacuum Deposition Organic Electronics [1]

**Table:** 

**Value of the Data** • A quick and easy morphological observation is important to evaluate thin-films with atomic thickness. • The presented visualization technique using a standard optical microscope can be used to optimize the experimental parameters for producing valuable thin-films. • The data indicates the effect of image processing and dark-field mode in optical microscopy for visualization. • The dataset for Raman measurement can be used to identification of the material. • The presented spectra can be used to check the presence of contamination or undesired organic.

## 1 Data

### 1.1 Optical microscopy

**Fig. 1:**
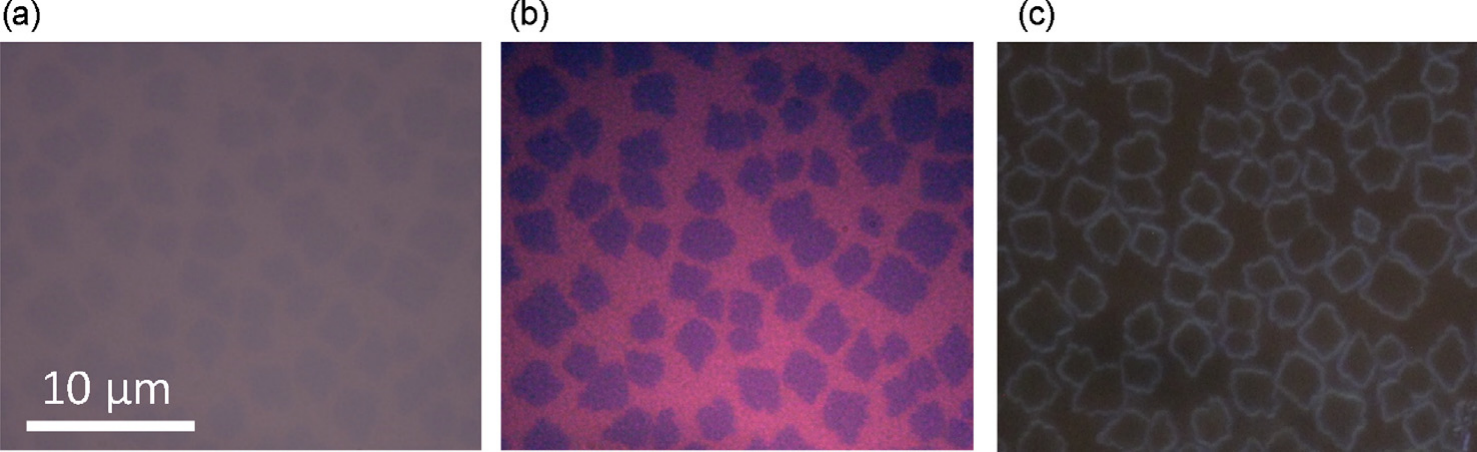
Comparison of the optical bright-field **(a, b)** and dark-field **(c)** microscopy images of monolayer 2D islands. The images **(a)** and **(c)** are the raw data without any enhancement. The color contrast in image **(b)** was strongly enhanced by image processing.

**Fig. 2:**
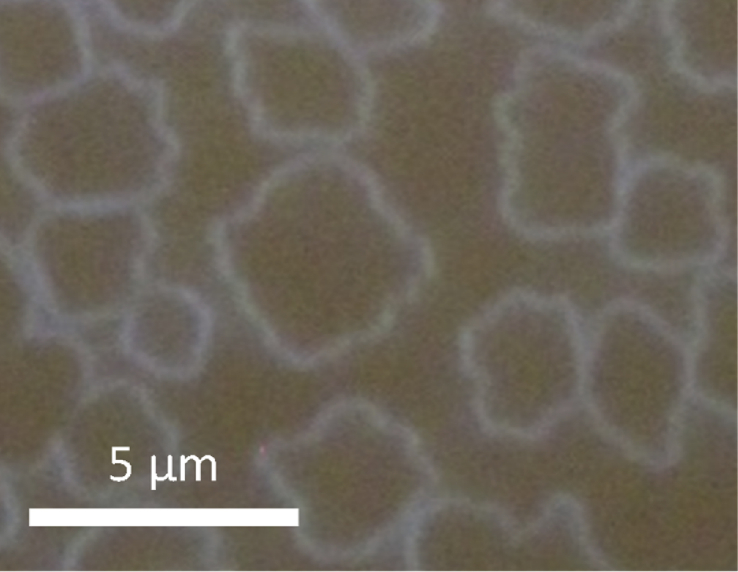
Optical dark-field microscopy image of large monolayer 2D islands. The film was deposited at 175 °C on a substrate treated with UV-O_3_. The nominal thickness of the films was 4.8 nm.

**Fig. 3:**
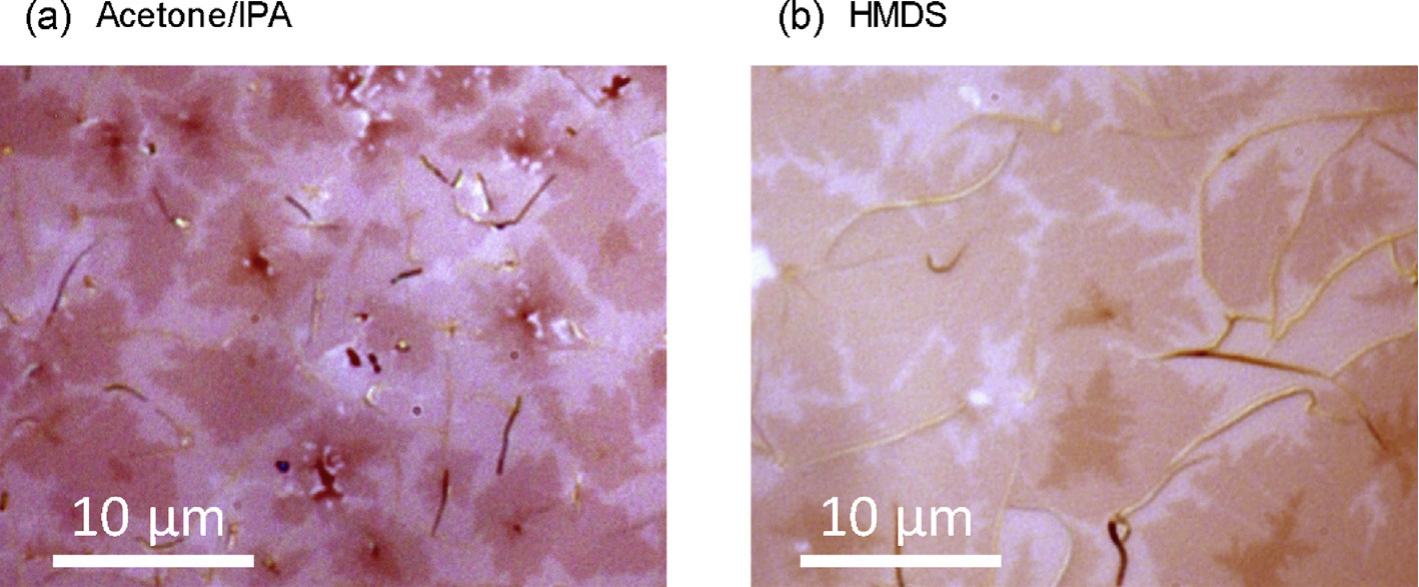
Optical bright-field microscopy images of the surface of the films deposited at 160 °C on the substrates cleaned with acetone/IPA **(a)** and treated with HMDS **(b)**. The nominal thickness of the films was 25 nm. Image color contrast was strongly enhanced by image processing. Although the size of 2D islands in the first layer on the substrates cleaned with acetone/IPA and treated with HMDS is small [1], large 2D islands with a fractal-like shape formed on the top layer. This suggests that the growth mechanism of subsequent layers after the first layer is not affected by substrate treatment.

Fig. 1, Fig. 2, Fig. 3 show optical microscopy images for 2,9-diphenyl-dinaphtho[2,3-*b*:2′,3′-*f*]thieno[3,2-b]thiophene (DPh-DNTT) thin-films prepared by vacuum deposition. Fig. 1, Fig. 2 show the microscopy images for monolayer two-dimensional (2D) islands on Si substrates with thermally grown 90-nm-thick SiO_2_. While, Fig. 3 shows the microscopy images for multilayer films.

### 1.2 Vibrational mode and Raman spectroscopy

**Fig. 4:**
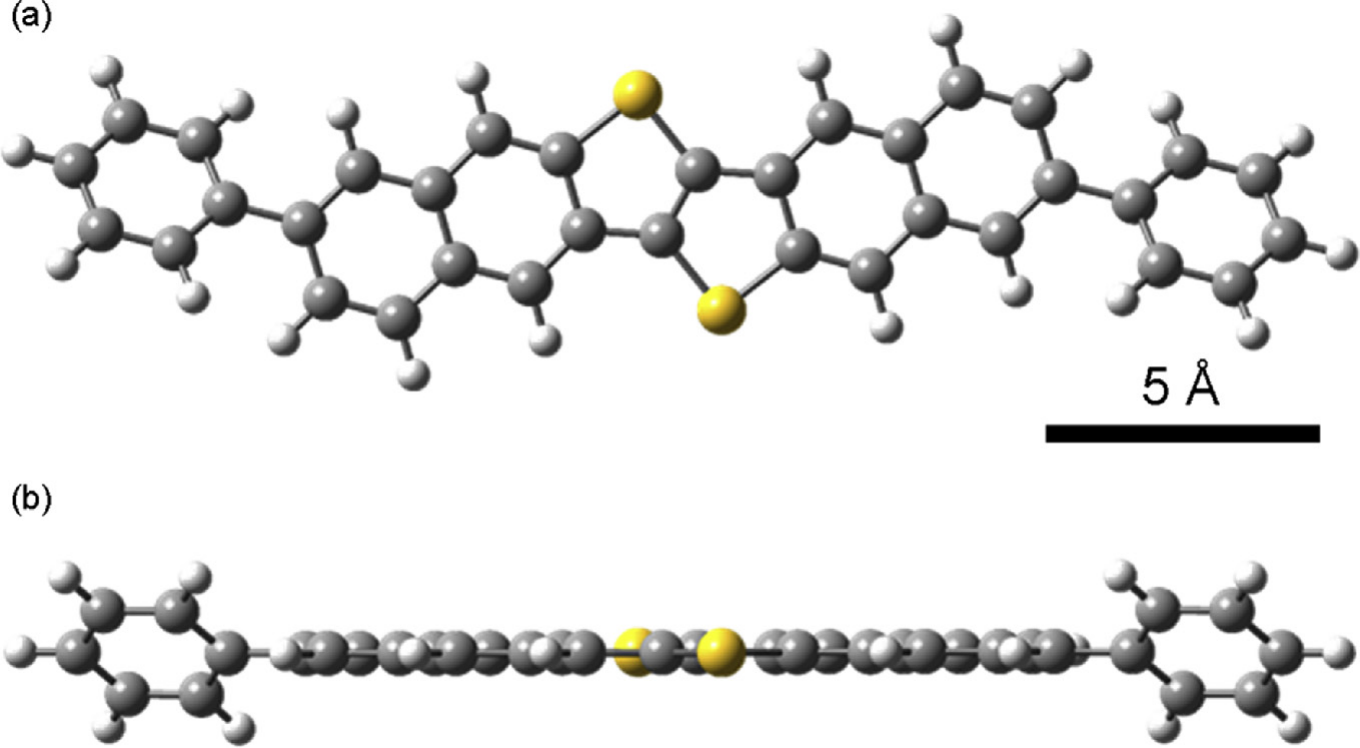
Structure of a single DPh-DNTT molecule as obtained by geometry optimization using quantum chemical calculations. The length in the longer molecular axis direction was ∼24.2 Å. **(b)** is different view of **(a)**.

**Fig. 5:**
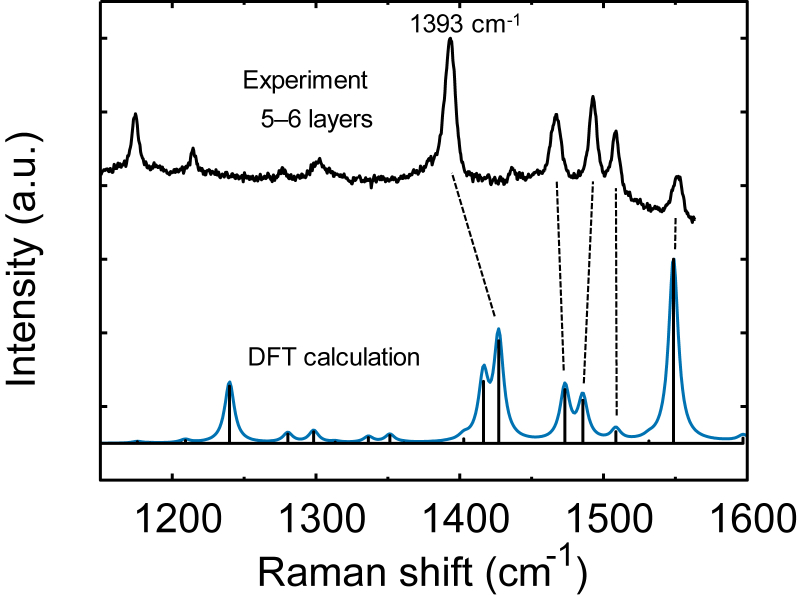
Comparison between measured Raman spectra of DPh-DNTT thin-film with 5 or 6 layers (black) and calculated vibrational mode (blue). Some vibrational modes correspond to the peaks observed in the Raman spectrum, which are indicated by the dotted lines in the figure.

**Fig. 6:**
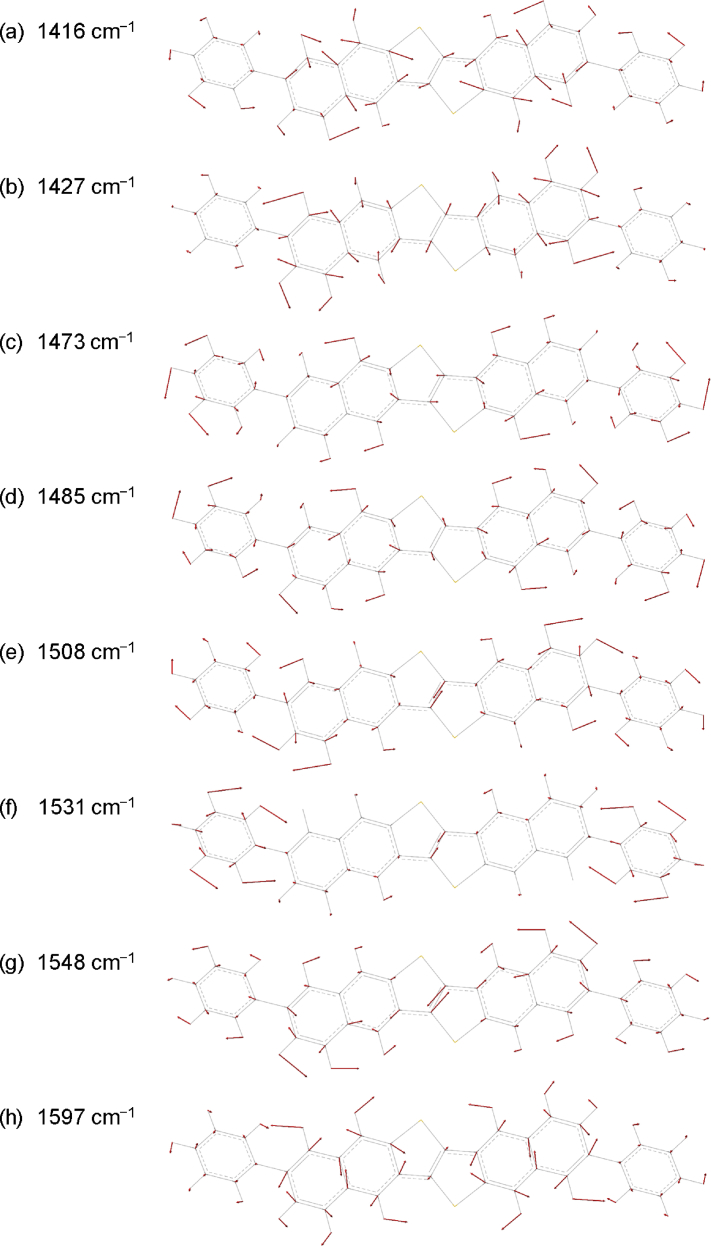
Vibrational modes of the peak at 1416 cm^−1^**(a)**, 1427 cm^−1^ **(b)**, 1473 cm^−1^**(c)**, 1485 cm^−1^ **(d)**, 1508 cm^−1^**(e)**, 1531 cm^−1^**(f)**, 1548 cm^−1^ **(g)**, and 1597 cm^−1^ **(h)**. The red arrows show the directions and the strength of displacements for each atom. The length of the arrows in all images was enhanced by the same factor for visualization and comparison.

Fig. 4, Fig. 5, Fig. 6 and Table 1 show quantum chemical calculations. The calculated vibrational modes were compared with the experimental Raman spectra in Fig. 5.

**Table 1:** Calculated vibrational modes.

Frequency (cm^−1^)	Intensity (a.u.)	Frequency (cm^−1^)	Intensity (a.u.)	Frequency (cm^−1^)	Intensity (a.u.)
32.8375	1.9019	865.5392	21.6165	1427.0560	8278.2309
50.2403	53.1690	891.8254	13.3087	1473.1367	4335.3717
67.7421	4.8659	916.0974	18.9164	1485.6043	3470.5150
110.2328	8.4114	917.4473	8.5209	1508.5654	943.1888
118.4318	48.3402	936.1403	2.6362	1531.5466	193.5745
138.9652	1.2429	954.0098	4.6950	1548.6475	14868.6764
191.7482	6.1354	979.1418	11.5196	1597.0006	420.8476
229.3356	3.0649	979.1485	0.0343	1623.1803	30.3989
277.1696	59.2242	983.6693	0.2704	1640.9157	17513.0983
310.3549	10.5493	983.6711	46.8939	1646.2203	0.0002
334.9245	3.9698	1001.6508	0.0031	1649.5227	3225.7920
369.3477	21.5759	1001.6613	2.3875	1665.1294	11133.2289
397.3028	65.8500	1013.2444	0.3957	3177.5742	0.2474
417.6818	97.8248	1013.2471	465.6890	3177.5799	49.2433
426.1558	5.8634	1040.1903	13.5363	3178.5413	0.1855
451.3575	3.0432	1061.6155	540.9385	3178.5516	97.4287
480.7578	16.4376	1069.1395	153.5213	3182.3190	3.3252
513.8443	69.3557	1106.1178	0.0008	3182.3326	185.8882
545.6701	169.4322	1106.1205	0.7717	3184.3113	121.1432
570.6369	1.1764	1175.6928	126.5441	3184.3362	1.9328
606.1332	17.2913	1184.5098	0.0406	3185.0914	2.4624
625.3482	22.9005	1184.5165	18.4967	3185.0983	180.3127
633.3783	12.0878	1206.5725	31.5363	3189.3452	0.0014
642.5825	3.2182	1209.1863	219.3229	3189.4493	229.5115
692.7496	51.5886	1239.7866	4609.2941	3193.9649	0.0216
705.4153	3.9558	1240.1679	0.0045	3193.9699	240.9489
709.9950	0.3325	1280.3649	743.3839	3199.2413	0.1081
731.0543	138.3137	1298.3726	926.1226	3199.2505	101.0743
761.0255	27.1047	1313.3467	97.5823	3201.9462	0.4853
773.5289	14.7745	1336.4099	464.6950	3201.9622	281.9536
798.5696	371.9362	1351.3435	631.8865	3207.7083	0.5069
825.4099	39.0921	1361.1393	28.4862	3207.7410	1093.9089
858.0423	82.7488	1402.7440	361.7260		
858.2422	0.0001	1416.4792	4999.0012		

## 2 Experimental design, materials, and methods

Quantum chemical calculations were performed using the Gaussian 09 program package [2] to compare the Raman spectra to the vibrational modes calculated for a DPh-DNTT molecule. The geometry optimization and vibrational analysis were performed using a hybrid density functional theory method combining the Becke's three-parameter exchange functional and the Lee-Yang-Parr's correlation functional (B3LYP) with the 6-31G+(d,p) basis set.
